# A Nationwide Assessment of the Burden of Urinary Tract Infection among Renal Transplant Recipients

**DOI:** 10.1155/2015/854640

**Published:** 2015-02-25

**Authors:** Benjamin J. Becerra, Monideepa B. Becerra, Nasia Safdar

**Affiliations:** ^1^School of Public Health, Loma Linda University, Loma Linda, CA 92350, USA; ^2^William S. Middleton Memorial Veterans Hospital, Madison, WI 53705, USA; ^3^Department of Health Science and Human Ecology, California State University San Bernardino, San Bernardino, CA 92407, USA; ^4^Department of Medicine, University of Wisconsin, Madison, WI 53706, USA

## Abstract

*Objective.* Evaluate the prevalence and outcomes of urinary tract infection (UTI) among renal transplant recipients. *Methods.* A secondary analysis of the Nationwide Inpatient Sample 2009–2011 was conducted. Survey-weighted multivariable regression analyses were used to examine the impact of UTI on transplant complications, total charges, and length of stay. *Results.* A total of 1,044 renal transplant recipients, representing a population estimate of 49,862, were included in the study. UTI was most common in transplant recipients with hypertension (53%) and prevalence was noted to be 28.2 and 65.9 cases per 1,000 for men and women, respectively. UTI increased the likelihood of transplant complications (182% for men, 169% for women). Total charges were 28% higher among men as compared to 22% among women with UTI. Such infection also increased the length of stay by 87% among men and 74% among women. *Discussion.* UTI in renal transplant recipients was associated with prolonged length of stay, total charges, and increased odds of transplant complications. Interventions to prevent UTI among such patients should be a priority area for future research and practice.

## 1. Introduction

Urinary tract infection (UTI) is the most common type of hospital-acquired infection (HAI) among renal transplant patients, surpassing viral infections, pneumonia, and surgical site infections [1, 2]. Renal transplant recipients are more likely to develop UTI as compared to the general population. Studies within the last decade have reported prevalence of such infection ranging from 13% to nearly 80% among renal transplant patients [3–9]. The classic signs and symptoms of UTI such as fever, urinary urgency, or abdominal pain may be lacking or attenuated in renal transplant recipients, due to immunosuppression [10].

While various risk factors of UTI have been evaluated in the literature, with consistency noted across studies on the heightened risk associated with increased age and female gender [4, 7, 8, 11], few studies have assessed the impact of UTI on patient or hospital outcomes. Much of the literature has reported graft dysfunction [9], graft loss [5, 8], and mortality [7, 8, 12], while other researchers noted no such findings [3]. To our knowledge, limited studies have evaluated the negative impact of UTI on both patient and health resource utilization among renal transplant recipients, especially using a nationally representative data source.

Such an evaluation is imperative, as the literature notes that while in nontransplant populations UTI may present with urgency, frequency, painful voiding, lower abdomen pain, and fever, symptoms may be lacking among transplant recipients, potentially due to the masking effect of immunosuppressive medications [10]. These results highlight the potential negative impact of UTI among other transplant patients, though limited studies have highlighted the burden among renal transplant patients. In this study, we addressed such a gap in the literature by utilizing a nationally representative survey and further evaluated the prevalence and impact of UTI on patient and hospital outcomes, including complications, length of stay, and total charges, among renal transplant recipients.

## 2. Methods

### 2.1. Data Source

We extracted data from 2009–2011 Nationwide Inpatient Sample (NIS), the largest publically available all-payer inpatient dataset in the United States. NIS is available yearly (since 1988) and includes data from all Agency for Healthcare Research and Quality (AHRQ) sponsored Healthcare Cost and Utilization Project (HCUP) participating states. Approximately, 8 million inpatient stays from 1,000 hospitals in the nation are included, reflective of a 20% sample of all nonfederal, short-term, general, and other specialty hospitals, except hospital units of institutions. Short-term rehabilitation (starting 1998), long-term acute care hospitals, psychiatric hospitals, and alcoholism/chemical dependency treatment facilities are excluded from NIS. Details of NIS data are described elsewhere [13].

**Figure 1:**
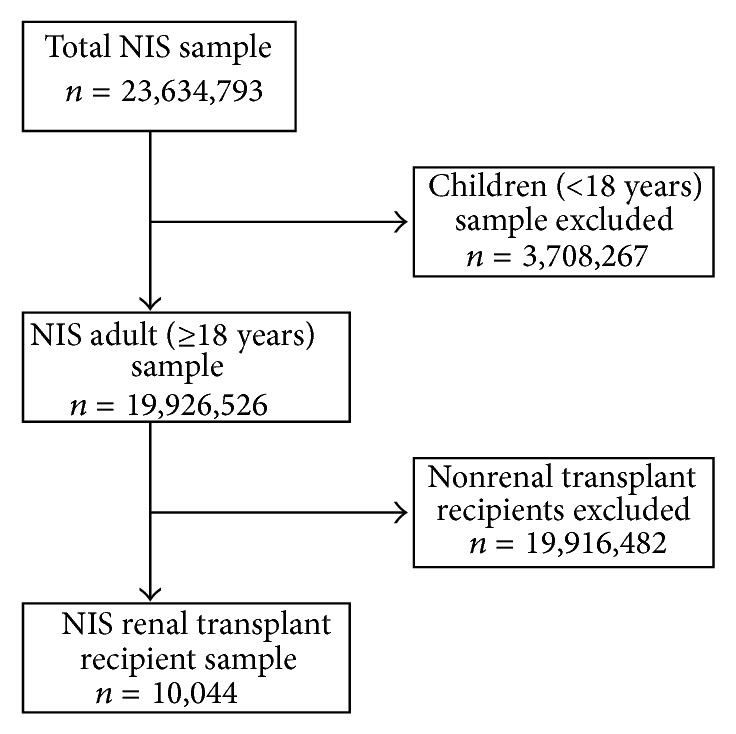
Sample selection procedure in study using Nationwide Inpatient Sample (NIS), 2009–2011.

In this study, our population was defined as those with a primary procedure code for renal transplant, resulting in a total of 10,044 discharges, representative of a population estimate of 49,862 recipients in the nation (average annual estimate = 16,621).  Figure 1 displays the sample selection procedure. Missing values were excluded from statistical analyses.

### 2.2. Measures

The primary exposure variable of this study was UTI diagnosis among renal transplant recipients. We used the International Classification of Diseases, 9th Revision, Clinical Modification (ICD-9-CM) Procedure codes of 55.6, 55.69, or ICD-9-CM Diagnosis code of V42.0 to identify renal transplant recipients. UTI was identified with ICD-9-CM code of 599.0.

The outcome variables were transplant complications, total charges, and length of stay. Transplant complications were defined as transplant failure or rejection (ICD-9-CM 996.81). Total charges and length of stay were both edited by AHRQ for uniformity between states. To account for inflation, we adjusted total charges to 2009 USD using the Gross Domestic Product (GDP) deflator from the United States Department of Commerce, Bureau of Economic Analysis [14].

Control variables comprised of both patient and hospital characteristics, in addition to survey year (2009, 2010, and 2011). Patient characteristics included donor type (live, deceased), age (18–34 years, 35–49 years, 50–64 years, 65 years or more), gender (men, women), race/ethnicity (White, Black, Hispanic, and others), median household income quartiles by patient ZIP code ($1–$38999, $39000–$47999, $48000–$62999, $63000 or more), primary payer type (Medicare, Medicaid, private including HMO, and others), and Charlson-Deyo index to take into account potential effect of other comorbid conditions.

The Charlson-Deyo index is a validated comorbidity measure for administrative data [15–17]. The index is comprised of 17 comorbidities, including myocardial infarction, congestive heart failure, peripheral vascular disease, cerebrovascular disease, dementia, chronic pulmonary disease, rheumatic disease, peptic ulcer disease, mild liver disease, diabetes with and without chronic complications, hemiplegia or paraplegia, renal disease, any malignancy (including lymphoma and leukemia, except malignant neoplasm of skin), moderate or severe liver disease, metastatic solid tumor, and human immunodeficiency virus/acquired immunodeficiency syndrome.

Hospital characteristics included bed size tertiles (small, medium, and large), hospital control or ownership (government nonfederal, private nonprofit, and private investor own), teaching status (teaching and nonteaching), and geographic location (Northeast, Midwest, South, and West).

### 2.3. Statistical Analyses

We conducted survey-weighted descriptive statistics to evaluate the prevalence of UTI among renal transplant recipients, in addition to the distribution of both patient and hospital characteristics in the entire sample, by gender. In order to assess the impact of UTI on complications of transplant among renal transplant recipients, we conducted chi-square tests followed by survey-weighted binary logistic regression analyses for both men and women. We used Wilcoxon rank sum to assess differences in total charges and length of stay among renal transplant recipients with UTI, as compared to those without. Finally, we conducted survey-weighted linear and negative binomial regressions to assess the impact of UTI on total charges and length of stay, respectively. In addition, log-transformation was employed for total charges due to nonnormality of such data.

Given that older adults are more likely to experience worse health and hospital outcomes due to a greater frequency of comorbid conditions [18–20], we conducted a sensitivity analysis to evaluate if the negative impact of UTI among renal transplant recipients was present among adults less than 65 years of age.

We used SAS 9.4 (SAS Institute, Inc., Cary, NC) for all statistical analyses except for negative binomial regression, for which we used STATA 12 package (Stata Corp LP, College Station, TX). Survey weights were applied in all statistical analyses, unless otherwise stated, in addition to design-based *F* values for variance estimation. To reduce rate of type I error due to multiple testing, we employed a Bonferroni familywise correction and *P* < 0.003 was used to denote significance for all statistical analyses. The STROBE checklist for observational studies was followed for reporting results.

## 3. Results

As summarized in Table 1, we found a prevalence of 28.2 and 65.9 cases of UTI per 1,000 for men and women, respectively, in the study population of renal transplant recipients. The majority of transplant patients received kidneys from deceased donors (60% men, 63% women), were aged 50–64 years (39% men, 38% women), were White (49% men, 47% women), had Medicare (59% men, 60% women), and were hospitalized in a teaching hospital (95% men, 96% women). While income and comorbidity distribution were nearly equal in all categories for both genders, the highest percent of renal transplants occurred in the Southern states (37% men, 38% women).

Rate of complications was higher for men who were renal transplant recipients with UTI as compared to those without (42% versus 19%, *P* < 0.0001). A similar trend was noted among women with UTI as compared to those without (38% versus 18%, *P* < 0.0001). Results of survey-weighted multivariable logistic regression analysis showed that renal transplant recipients with UTI were nearly three times more likely to have complications of transplant (adjusted odds ratio (aOR) men = 2.8; aOR women = 2.6), as compared to those without UTI (Table 2).

UTI was also associated with increased total charges for both men ($549,659 versus $335,711, *P* < 0.0001) and women ($509,714 versus $335,140, *P* < 0.0001). Results of survey-weighted multiple linear regression analyses (Table 3) showed that UTI was associated with significantly higher total charges among both men (24%) and women (22%).

Renal transplant recipients with UTI had increased length of stay as compared to those without such infection (10 days versus 6 days for men, *P* < 0.0001; 9 days versus 6 days for women, *P* < 0.0001). Negative binomial regression results demonstrated that UTI was associated with increases in length of stay for men (adjusted incidence rate ratio (aIRR) = 1.9) and women (aIRR = 1.7) renal transplant recipients, respectively (Table 4).

We also found gender differences on the burden of UTI among such transplant recipients. For example, men were 182% more likely while women were 160% more likely to have complications upon UTI (Table 2). Similarly, total charges were 28% higher among men as compared to 22% among women with UTI (Table 3). Finally, UTI increased the likelihood of length of stay among men by 87% as compared to 74% among women (Table 4).

Several other characteristics were associated with increased complications, charges, or length of stay among such transplant patients. Organ donation from deceased donor substantially increased the likelihood of transplant complications (aOR men = 2.3; aOR women = 1.9), percent increase in total charges (men = 17.9; women = 18.9), and length of stay (aIRR = 1.3 for men and women). Having Medicare was related to increased odds of transplant complications (aOR men = 1.3; aOR women = 1.5) and length of stay (aIRR = 1.1 for men and women). Other patient and hospital characteristics associated with increased total charges were having three or more comorbidities, teaching status of hospital, and increasing calendar year. Increased length of stay was also significantly associated with three or more comorbidities and having Medicaid (for men only).

Sensitivity analyses further demonstrated that the negative impact of UTI remained significant among adult renal transplant recipients aged 64 years or less (Table 5). UTI was significantly associated with transplant complications among both men (aOR = 3.45) and women (aOR = 2.68). Similarly, men and women renal transplant patients with UTI had nearly 30% and 22% higher total charges, as compared to those with no such infection. We also found that, compared to those without UTI, length of stay was 74% and 76% higher among men and women renal transplant recipients, respectively, with UTI.

## 4. Discussion

Using a large nationally representative sample, we found that UTI remains a significant complication among renal transplant patients and is associated with considerable negative outcomes in this patient population. We found that risks of transplant complications (transplant failure or rejection) were significantly higher for both men and women renal transplant recipients with UTI, as were increased rates of health resource utilization (total charges and length of stay).

Previous studies of UTI in renal transplant recipients have shown that both early and late UTI may have serious adverse consequences in this population. While some of these studies have noted increased hospital days and costs related to catheter-associated UTI [21, 22], few to date have evaluated complications and health resource utilization related to UTI among renal transplant recipients. For example, Abbott and colleagues undertook a retrospective cohort study of 28,942 Medicare primary renal transplant recipients in the United States Renal Data System database from 1996 through 2000, assessing Medicare claims for UTI occurring later than 6 months after transplantation based on ICD-9 codes, and found that the cumulative incidence of UTI during the first 6 months after renal transplantation was 17% (equivalent for both men and women) and at 3 years was 60% for women and 47% for men (*P* < 0.001 in Cox regression analysis). Late UTI was significantly associated with an increased risk of subsequent death and graft loss. Our study extends the literature in this area by using the largest national inpatient sample which is not limited to Medicare claims [8].

In a retrospective analysis on 500 adult renal transplant recipients at two transplant centers in the United States, Chuang et al. [7] found that, within an average follow-up period of 42 months, approximately 43% of the transplant recipients developed UTI. While UTI did not significantly impact graft loss, it greatly increased mortality among such patients. In our study, we noted a similar negative impact on transplant complications among kidney recipients with UTI, though our results expanded beyond two national facilities and included a nationally representative sample.

In a study utilizing the United States Renal Data System database, Dhamidharka et al. [23] analyzed 1996–2000 Medicare claims to evaluate the impact of UTI 36 months after kidney transplant among 870 children. The authors found that early UTI (less than 6 months after transplant) was significantly (*P* = 0.007 upon multivariable Cox regression) associated with higher adjusted hazard ratio of graft loss, and late UTI was not associated with such outcome. The researchers further noted no significant association between UTI (early or late) and mortality. The results from our study on transplant complications, length of stay, and total charges demonstrate that potential postoperative UTI could not only have significant impact patient outcomes, but also increase health resource utilization and further burden the healthcare system. Such results, thus, highlight the need for monitoring UTI among renal transplant recipients to ensure positive outcomes.

Other researchers evaluating the complications of UTI on various patient populations have further reported the negative impacts. For example, Kang et al. [24] noted that, among colorectal cancer surgery patients, UTI was significantly associated with increased length of stay and hospital charges, as compared to those without UTI. Similarly, UTI has been associated with increased length of stay and hospital costs in general and major elective surgery patients [21, 25, 26]. Cumulatively, the current empirical evidence notes a negative effect of UTI after surgery and/or transplantation. Our results further add to the limited body of literature on the complications and health resource utilization associated with UTI among adult renal transplant recipients.

In addition, we found gender differences in the adverse outcomes of UTI, with men reporting higher rates of complications, higher charges, and length of stay; and reasons for these differences should be explored in future studies. Several other factors, such as deceased donor, increased age, Medicare, and presence of several comorbidities, were significantly associated with negative outcomes in renal transplant recipients. Such results are consistent with previous studies that have shown increased risk of complications or mortality to be associated with use of deceased donor, increased age, or presence of other comorbidities among other patients [23, 27–29]. Given the relation to increased age and such outcomes, the association with Medicare was expected. Increased length of stay among men renal transplant recipients with Medicaid, however, highlights the excess burden among low-income men as Medicaid is intended primarily for individuals of low socioeconomic status.

The results of our study should be interpreted in the context of some limitations. The unit of analysis in NIS is discharge and thus evaluation of repeat patients and individual-level variables could not be assessed. Our goal, however, in this study was to evaluate the burden of UTI among renal transplant hospitalizations, and such NIS provides an ideal data for such evaluation, though further studies on patient-level data are warranted. In addition, the role of other patient-level determinants, such as health literacy, education, and past experience in healthcare system, could not be evaluated. We also did not have data on the microbiology of UTI or treatment variables such as antibiotic use.

Our study also has a number of strengths. The use of a nationally representative sample ensures better variance estimations and thus results are generalizable to the United States population. Moreover, NIS includes a variety of payers including public and private and those who are uninsured, a significant strength as compared to other databases such as Medicare claims [30]. Our study of the NIS database highlights the prevalence and major negative consequences of UTI among renal transplant recipients. Given such outcomes, research to identify efficacious strategies to prevent UTI in kidney transplant recipients is needed.

## Figures and Tables

**Table 1:** Characteristics of study population (*n*, weighted %), NIS 2009–2011 (*n* = 10,044; *N* = 49,862).

	Men	Women
Urinary tract infection	173 (2.82)	262 (6.59)
Donor type		
Deceased	3,650 (60.19)	2,467 (62.53)
Live	2,236 (36.44)	1,351 (34.11)
Missing	204 (3.37)	132 (3.35)
Age (years)		
18–34	820 (13.38)	640 (16.23)
35–49	1,829 (29.93)	1,167 (29.42)
50–64	2,390 (39.26)	1,515 (38.37)
65 or more	1,051 (17.42)	628 (15.98)
Race/ethnicity		
White	2,999 (49.32)	1,864 (47.26)
Black	1,313 (21.59)	936 (23.59)
Hispanic	887 (14.64)	562 (14.31)
Others	455 (7.51)	317 (8.20)
Missing	436 (6.93)	271 (6.64)
Charlson-Deyo index		
Two or less	2,993 (49.09)	1,913 (48.63)
Three or more	3,097 (50.91)	2,037 (51.37)
Neighborhood income		
$1–$38,999	1,595 (26.04)	1,132 (28.50)
$39,000–$47,999	1,475 (24.16)	939 (23.81)
$48,000–$62,999	1,527 (25.25)	952 (24.16)
$63,000 or more	1,351 (22.22)	841 (21.34)
Missing	142 (2.34)	86 (2.18)
Payer type		
Private including HMO	2,021 (33.03)	1,253 (31.66)
Medicare	3,589 (59.06)	2,370 (60.02)
Medicaid	201 (3.36)	154 (3.96)
Others	215 (3.46)	129 (3.19)
Missing	64 (1.08)	44 (1.16)
Teaching status		
Nonteaching	172 (2.65)	80 (1.94)
Teaching	5,801 (95.46)	3,799 (96.30)
Missing	117 (1.89)	71 (1.76)
Geographic location		
Northeast	1,073 (17.99)	621 (15.97)
Midwest	1,512 (23.86)	1,000 (24.35)
South	2,250 (36.54)	1,508 (37.75)
West	1,255 (21.61)	821 (21.93)
Year		
2009	2,201 (35.81)	1,411 (35.48)
2010	1,944 (33.76)	1,245 (33.37)
2011	1,945 (30.43)	1,294 (31.16)

**Table 2:** Multivariable logistic regression odds ratio (and 95% CI) for complications of transplant among renal transplant recipients, NIS 2009–2011.

	Men		Women	
	OR (95% CI)	*P* value	OR (95% CI)	*P* value
UTI versus no UTI	2.82 (1.90, 4.18)	<0.001^*^	2.60 (1.89, 3.58)	<0.001^*^
Deceased donor versus live	2.29 (1.92, 2.73)	<0.001^*^	1.87 (1.45, 2.41)	<0.001^*^
Age (Ref. = 18–34 years)				
35–49 years	0.97 (0.81, 1.16)	0.71	0.99 (0.73, 1.35)	0.94
50–64 years	0.83 (0.68, 1.02)	0.08	0.84 (0.61, 1.15)	0.27
65 years or more	0.80 (0.59, 1.09)	0.15	0.60 (0.42, 0.87)	0.01
Race/ethnicity (Ref. = White)				
Black	0.89 (0.74, 1.07)	0.20	0.86 (0.70, 1.06)	0.16
Hispanic	0.97 (0.77, 1.22)	0.80	0.90 (0.69, 1.17)	0.42
Others	0.84 (0.60, 1.17)	0.30	0.70 (0.49, 1.00)	0.05
Charlson-Deyo index 3 or more versus 2 or less	0.90 (0.78, 1.04)	0.15	0.83 (0.68, 1.02)	0.07
Neighborhood income (Ref. = $1–$38,999)				
$39,000–$47,999	1.14 (0.94, 1.37)	0.18	1.20 (0.92, 1.55)	0.18
$48,000–$62,999	1.01 (0.80, 1.28)	0.93	1.41 (1.11, 1.80)	0.005
$63,000 or more	1.20 (0.95, 1.51)	0.13	1.36 (1.05, 1.75)	0.02
Payer type (Ref. = private including HMO)				
Medicare	1.31 (1.11, 1.55)	0.001^*^	1.51 (1.27, 1.81)	<0.001^*^
Medicaid	1.38 (0.96, 1.98)	0.09	1.21 (0.75, 1.97)	0.43
Others	0.43 (0.19, 0.99)	0.05	1.23 (0.60, 2.51)	0.57
Teaching versus nonteaching	1.21 (0.87, 1.68)	0.25	1.40 (0.57, 3.47)	0.46
Geographic location (Ref. = Northeast)				
Midwest	0.84 (0.48, 1.48)	0.55	0.71 (0.49, 1.04)	0.08
South	0.73 (0.45, 1.17)	0.19	0.59 (0.41, 0.85)	0.004
West	0.97 (0.62, 1.51)	0.88	0.59 (0.39, 0.90)	0.01
Year (Ref. = 2009)				
2010	0.89 (0.63, 1.25)	0.50	1.11 (0.85, 1.46)	0.44
2011	1.04 (0.72, 1.50)	0.85	1.17 (0.87, 1.58)	0.30

^*^Bonferroni *P* < 0.003.

Ref. = reference category, UTI = urinary tract infection, OR = odds ratio, and CI = confidence interval.

**Table 3:** Multiple linear regression for percent change in total charges in dollars (and 95% CI) among renal transplant recipients, NIS 2009–2011.

	Men		Women	
	Percent change (95% CI)	*P* value	Percent change (95% CI)	*P* value
UTI versus no UTI	28.43 (17.49, 39.38)	<0.001^*^	22.07 (14.38, 29.76)	<0.001^*^
Deceased donor versus live	17.89 (12.46, 23.32)	<0.001^*^	18.75 (12.24, 25.26)	<0.001^*^
Age (Ref. = 18–34 years)				
35–49 years	−1.77 (−6.26, 2.72)	0.44	−2.08 (−6.57, 2.41)	0.36
50–64 years	−5.89 (−10.53, −1.26)	0.01	−5.31 (−9.79, −0.83)	0.02
65 years or more	−4.70 (−10.01, 0.61)	0.08	−5.95 (−12.00, 0.09)	0.05
Race/ethnicity (Ref. = White)				
Black	8.50 (1.52, 15.49)	0.02	5.26 (−2.20, 12.73)	0.17
Hispanic	2.51 (−6.78, 11.79)	0.60	5.41 (−4.83, 15.65)	0.30
Others	−4.68 (−13.16, 3.80)	0.28	−5.79 (−13.56, 1.97)	0.14
Charlson-Deyo index 3 or more versus 2 or less	8.43 (5.72, 11.13)	<0.001^*^	10.67 (7.44, 13.90)	<0.001^*^
Neighborhood income (Ref. = $1–$38,999)				
$39,000–$47,999	−0.47 (−5.01, 4.08)	0.84	−0.45 (−5.62, 4.72)	0.86
$48,000–$62,999	−0.99 (−6.45, 4.47)	0.72	1.91 (−4.57, 8.39)	0.56
$63,000 or more	−0.59 (−8.74, 7.57)	0.89	1.22 (−9.23, 11.68)	0.82
Payer type (Ref. = private including HMO)				
Medicare	6.05 (1.75, 10.34)	0.01	7.83 (2.59, 13.07)	0.003
Medicaid	17.91 (4.28, 31.54)	0.01	14.00 (0.17, 27.83)	0.05
Others	7.19 (−7.68, 22.07)	0.34	3.39 (−15.40, 22.18)	0.72
Teaching versus nonteaching	−43.74 (−61.71, −25.77)	<0.001^*^	−43.99 (−58.62, −29.35)	<0.001^*^
Geographic location (Ref. = Northeast)				
Midwest	15.96 (−5.46, 37.37)	0.14	13.85 (−6.69, 34.40)	0.19
South	−8.95 (−30.53, 12.63)	0.42	−9.85 (−30.03, 10.32)	0.34
West	10.93 (−16.23, 38.08)	0.43	11.17 (−13.91, 36.25)	0.38
Year (Ref. = 2009)				
2010	94.38 (80.54, 108.23)	<0.001^*^	94.51 (81.51, 107.50)	<0.001^*^
2011	159.01 (144.82, 173.20)	<0.001^*^	157.94 (144.51, 171.36)	<0.001^*^

^*^Bonferroni *P* < 0.003.

Ref. = reference category, UTI = urinary tract infection, and CI = confidence interval.

**Table 4:** Multivariable negative binomial incidence rate ratio (and 95% CI) for length of stay among renal transplant recipients, NIS 2009–2011.

	Men		Women	
	IRR (95% CI)	*P* value	IRR (95% CI)	*P* value
UTI versus no UTI	1.87 (1.50, 2.33)	<0.001^*^	1.74 (1.55, 1.97)	<0.001^*^
Deceased donor versus live	1.31 (1.24, 1.39)	<0.001^*^	1.26 (1.16, 1.37)	<0.001^*^
Age (Ref. = 18–34 years)				
35–49 years	1.06 (1.00, 1.12)	0.05	0.95 (0.88, 1.02)	0.15
50–64 years	1.00 (0.93, 1.07)	0.95	0.93 (0.86, 0.99)	0.03
65 years or more	1.15 (1.03, 1.28)	0.01	0.98 (0.90, 1.07)	0.64
Race/ethnicity (Ref. = White)				
Black	1.07 (0.99, 1.16)	0.07	1.02 (0.95, 1.10)	0.53
Hispanic	0.99 (0.90, 1.07)	0.76	1.01 (0.92, 1.11)	0.84
Others	0.96 (0.88, 1.04)	0.32	0.93 (0.85, 1.01)	0.09
Charlson-Deyo index 3 or more versus 2 or less	1.16 (1.11, 1.21)	<0.001^*^	1.16 (1.11, 1.22)	<0.001^*^
Neighborhood income (Ref. = $1–$38,999)				
$39,000–$47,999	0.99 (0.93, 1.05)	0.71	0.99 (0.92, 1.06)	0.74
$48,000–$62,999	1.00 (0.93, 1.08)	0.98	1.01 (0.93, 1.11)	0.74
$63,000 or more	0.97 (0.88, 1.07)	0.54	1.02 (0.90, 1.16)	0.71
Payer type (Ref. = private including HMO)				
Medicare	1.11 (1.05, 1.17)	<0.001^*^	1.11 (1.05, 1.17)	<0.001^*^
Medicaid	1.21 (1.09, 1.35)	<0.001	1.21 (1.03, 1.43)	0.02
Others	1.03 (0.91, 1.17)	0.66	1.23 (0.94, 1.63)	0.13
Teaching versus nonteaching	0.81 (0.69, 0.95)	0.01	0.78 (0.67, 0.93)	0.01
Geographic location (Ref. = Northeast)				
Midwest	0.94 (0.77, 1.14)	0.51	0.95 (0.78, 1.16)	0.63
South	0.97 (0.82, 1.15)	0.71	0.98 (0.80, 1.20)	0.85
West	1.02 (0.87, 1.18)	0.83	1.03 (0.86, 1.22)	0.77
Year (Ref. = 2009)				
2010	1.01 (0.89, 1.16)	0.85	1.05 (0.93, 1.19)	0.44
2011	1.04 (0.90, 1.20)	0.63	1.04 (0.89, 1.23)	0.61

^*^Bonferroni *P* < 0.003.

Ref. = reference category, UTI = urinary tract infection, IRR = incidence rate ratio, and CI = confidence interval.

**Table 5:** Multivariable regression analyses of sensitivity analysis (excluding 65 years or older) on impact of urinary tract infection among renal transplant recipients, NIS 2009–2011^a^.

	Transplant complications	Total charges	Length of stay
	OR (95% CI)	Percent change (95% CI)	IRR (95% CI)
Men	3.45 (2.62, 5.26)^*^	29.51 (18.32, 40.70)^*^	1.74 (1.51, 1.99)^*^
Women	2.68 (1.92, 3.73)^*^	21.69 (12.67, 30.70)^*^	1.76 (1.56, 1.97)^*^

^*^Bonferroni *P* < 0.003.

^a^Model adjusted for age, donor type, race/ethnicity, Charlson-Deyo index, neighborhood income, payer type, teaching status, geographic location of hospital, and year.

OR = odds ratio, CI = confidence interval, and IRR = incidence rate ratio.
